# Exploring the Role of the LCN2–DHODH Interaction in Mitochondrial Ferroptosis During Sepsis-Induced Myocardial Injury and LPS-Induced HL-1 Cardiomyocyte Injury

**DOI:** 10.3390/ijms27156972

**Published:** 2026-08-03

**Authors:** Lu Li, Yuping Li, Yixuan Hao, Mengjie Yu, Shicheng Xia, Jiahui Wang, Hongwei Ye, Qin Gao

**Affiliations:** 1 Department of Physiology, Bengbu Medical University, Bengbu 233000, China; lilu52761004@163.com (L.L.); 16632832991@163.com (Y.H.); 2 Key Laboratory of Basic and Clinical Cardiovascular Diseases, Bengbu Medical University, Bengbu 233000, China; 16655230731@163.com (Y.L.); ymj11711@163.com (M.Y.); wangjh202202@126.com (J.W.); 3 School of Life Sciences, Bengbu Medical University, Bengbu 233000, China; 4 Department of Clinical Medicine, Bengbu Medical University, Bengbu 233000, China; 12210110342@stu.bbmc.edu.cn

**Keywords:** sepsis induce myocardial injury, mitochondrial derived ferroptosis, lipocalin-2, dihydroorotate dehydrogenase, STAT3

## Abstract

This study aimed to investigate the regulatory effect of lipocalin-2 (LCN2) knockdown on DHODH-mediated mitochondrial ferroptosis in sepsis-induced myocardial injury (SIMI) and clarify the underlying STAT3-related molecular mechanism in a cecal ligation and puncture (CLP) mouse model and lipopolysaccharide (LPS)-stimulated HL-1 cardiomyocyte injury model. We established a classic cecal ligation and puncture (CLP)-induced SIMI mouse model. Pharmacological suppression of ferroptosis was performed to confirm the pathogenic role of ferroptosis in SIMI progression. Subsequently, LCN2-knockdown mice were utilized to explore the biological function of LCN2 in modulating myocardial ferroptosis and septic cardiac injury, and the direct protein interaction between LCN2 and DHODH was verified via molecular docking and co-immunoprecipitation assays. In vitro, we constructed stable DHODH-overexpressing HL-1 cardiomyocytes via lentiviral transfection and established a lipopolysaccharide (LPS)-induced cardiomyocyte injury model. The results showed that LCN2 expression was markedly upregulated in SIMI. Both GPX4- and DHODH-dependent mitochondrial ferroptosis were significantly activated during SIMI. Lentivirus-mediated DHODH overexpression exerted prominent protective effects against LPS-induced cardiomyocyte injury. Importantly, pharmacological blockade of DHODH by Brequinar reversed the reduction of p-STAT3 induced by LCN2 silencing. However, Fin56-mediated specific inhibition of mitochondrial GPX4 exhibited no significant effect on STAT3 phosphorylation level, revealing distinct regulatory mechanisms for these two pathways. In summary, LCN2 knockdown inhibits DHODH-mediated mitochondrial ferroptosis to alleviate SIMI. Pharmacological inhibition of DHODH abrogates the cardioprotective effect of LCN2 knockdown by restoring STAT3 phosphorylation. These findings provide novel insights into the prevention and treatment of sepsis-induced myocardial injury.

## 1. Introduction

Sepsis is a life-threatening multiple organ dysfunction syndrome triggered by dysregulated host inflammatory responses to infection accompanied by simultaneous hyperinflammation and immunosuppression and contributes to substantial global morbidity and mortality [1]. Sepsis-induced myocardial injury (SIMI), also defined as septic cardiomyopathy, is one of the most devastating complications of sepsis, characterized by myocardial inflammation, hypotension, arrhythmia, and cardiac dysfunction [2]. Accumulating clinical evidence confirms that SIMI is a predominant contributor to sepsis-associated mortality, severely compromising the long-term prognosis of septic patients [3].

Ferroptosis, an iron-dependent non-apoptotic programmed cell death, has been increasingly recognized as a critical driver of sepsis-related multi-organ injury. Distinct from apoptosis and necrosis, ferroptosis is initiated by disrupted cellular redox homeostasis, excessive iron accumulation, and uncontrolled lipid peroxidation, leading to massive phospholipid hydroperoxide (PLOOH) and reactive oxygen species (ROS) accumulation. Its morphological features include mitochondrial shrinkage, increased membrane density, and reduction or disappearance of mitochondrial cristae [4]. As the organelle most sensitive to cellular injury, the mitochondrion plays a central role in iron and energy metabolism and represents a major source of intracellular ROS, thus being closely associated with ferroptosis [5]. Emerging studies have validated the pivotal role of mitochondrial ferroptosis in the progression of SIMI, making it a promising therapeutic target for septic cardiac dysfunction. Pharmacological blockade of ferroptosis using Ferrostatin-1 (Fer-1) effectively rescues mitochondrial abnormalities and alleviates myocardial damage in septic models, while dysregulated cardiac glutathione metabolism and GPX4 depletion further exacerbate cardiomyocyte ferroptosis and mitochondrial lesions [6,7,8,9]. Dihydroorotate dehydrogenase (DHODH) is a mitochondrial rate-limiting enzyme responsible for de novo pyrimidine synthesis, which functionally couples pyrimidine biosynthesis with the mitochondrial electron transport chain [10]. Traditionally, DHODH has been extensively investigated as a therapeutic target for multiple malignancies [11,12,13,14,15,16]. Recent studies have revealed the regulatory effects of DHODH on ferroptosis in tumors [10]; however, its functional role and molecular mechanism in modulating mitochondrial ferroptosis during SIMI remain poorly elucidated.

Lipocalin-2 (LCN2) is an iron-binding secretory protein that participates in innate immunity and iron metabolic homeostasis. LCN2 is minimally expressed in normal cardiac tissues but is markedly upregulated under inflammatory and pathological stress [17,18,19,20]. Functionally, LCN2 participates in cardiovascular inflammatory responses by modulating the expression and secretion of pro-inflammatory cytokines and cell adhesion molecules, thereby driving endothelial inflammation and vascular injury [21,22]. Moreover, elevated LCN2 expression is closely correlated with the progression of various cardiac disorders, including heart failure, myocardial ischemia–reperfusion injury, coronary artery disease, and myocardial infarction [23,24,25]. Consistently, hypoxic stress significantly upregulates LCN2 expression in HL-1 cardiomyocytes, and increased LCN2 levels are also detectable in rodent hearts after transplantation [24,26]. These collective findings indicate that LCN2 serves as a reliable biomarker for evaluating the severity and poor prognosis of multiple cardiac diseases.

As a transporter which regulates intracellular and extracellular iron levels, LCN2 directly controls iron metabolism. Bacterial growth and metabolism require iron, and invading pathogens utilize iron-binding proteins to sequester iron from host transferrin and lactoferrin [27,28]. Research indicates that LCN2 promotes lipid peroxidation accumulation by increasing intracellular Fe^2+^, thereby exacerbating ferroptosis [29]. Our previous work has confirmed aberrant LCN2 upregulation in CLP-induced myocardial injury, which correlates with disordered iron metabolism and enhanced ferroptosis [30,31]. Nevertheless, the precise mechanism by which LCN2 modulates mitochondrial ferroptosis in SIMI remains to be fully clarified.

Signal transducer and activator of transcription 3 (STAT3) is a core inflammatory signaling molecule that mediates cellular responses to inflammatory stress, hypoxia, and ischemic injury. Cytoplasmic STAT3 undergoes phosphorylation and subsequent nuclear translocation to activate downstream transcriptional programs. Aberrant STAT3 activation has been reported to promote cellular ferroptosis, and LCN2 acts as a pro-inflammatory upstream regulator of STAT3 signaling [32,33]. Although recent studies have demonstrated that LCN2 inhibition ameliorates SIMI by mitigating inflammation, mitochondrial abnormalities, and cardiomyocyte ferroptosis [34,35,36], the underlying molecular linkage among LCN2, STAT3, and mitochondrial ferroptosis remains unclear. In the present study, we systematically investigated the regulatory mechanism of LCN2 in SIMI by focusing on DHODH-mediated mitochondrial ferroptosis. Using LCN2-knockdown mice and LPS-stimulated HL-1 cardiomyocytes, combined with pharmacological intervention of DHODH and mitochondrial GPX4, DHODH overexpression, and exogenous recombinant LCN2 stimulation, we clarified the distinct regulatory effects of DHODH and GPX4 on STAT3 phosphorylation. This study identifies a novel LCN2/DHODH/STAT3 mitochondrial ferroptosis axis, providing an innovative mechanistic basis and therapeutic target for SIMI treatment.

## 2. Results

### 2.1. Myocardial LCN2 Expression Was Significantly Elevated During the Early Stage of Sepsis-Induced Myocardial Injury

**Figure 1:**
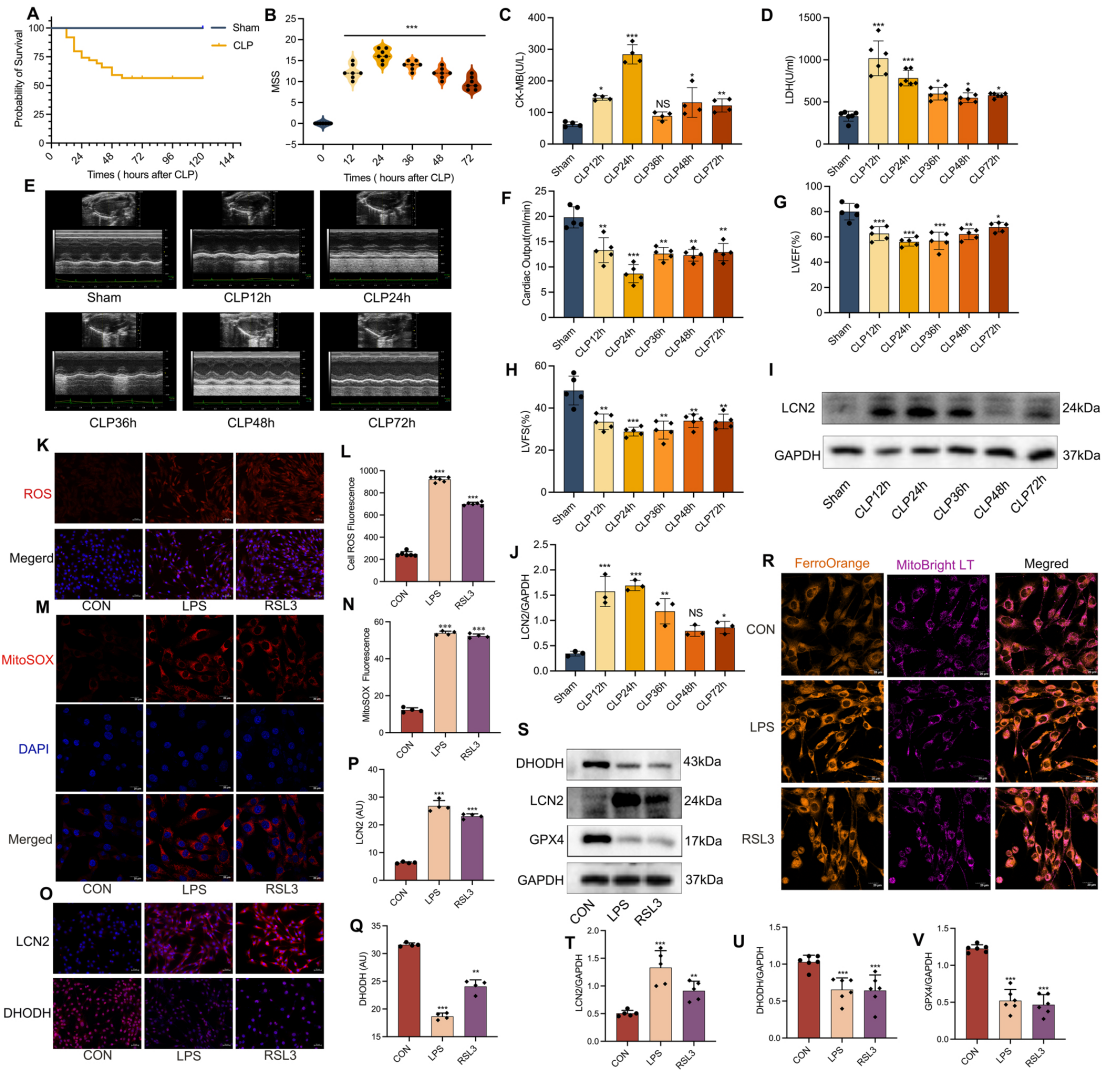
Elevated LCN2 level was associated with ferroptosis. (**A**): Survival curves of cecal ligation and puncture (**B**): Murine sepsis score (MSS). (**C**,**D**): Serum CK-MB and LDH levels in each group. (**E**): Evaluation of cardiac function by echocardiography. Representative M-mode images are shown. (**E**,**F**–**H**): Echocardiographic parameters. Cardiac output (**F**); left ventricle ejection fraction (**G**); left ventricular fractional shortening. (**H**,**I**,**S**): Representative images of the Western blot results. Changes in the protein expressions of LCN2 (**J**,**T**) (*n* = 3–6), GPX4 (**V**) and DHODH (**U**) (*n* = 6). (**K**): Representative images of fluorescence probe for ROS in HL-1 cardiomyocytes (*n* = 6, scale bar = 50 μm). (**L**): The ROS fluorescence in each group. (**M**): Images of mitochondrial ROS in different groups. (**N**): Mitochondrial ROS fluorescence intensity analysis. (*n* = 5, scale bar = 20 μm). (**O**): Images of LCN2 and DHODH (red) and DAPI (blue) immunofluorescence staining. (**P**,**Q**): LCN2 and DHODH fluorescence intensity analysis. (n = 6, scale bar = 50 μm). (**R**): Detection of intracellular Fe^2+^ and mitochondrial morphology of HL-1 cardiomyocytes in different groups (scale bar = 20 μm). Data are presented as mean ± SD. *** *p* < 0.001, ** *p* < 0.01, * *p* < 0.05 vs. Sham group, NS: *p* > 0.05 vs. Sham group. *** *p* < 0.001, ** *p* < 0.01 vs. CON group.

We established a mouse sepsis model via CLP surgery. The severity of sepsis was assessed using the MSS at 12, 24, 36, 48 and 72 h after surgery [37]. As shown in Figure 1A,B, myocardial injury reached its peak at 12 h and 24 h following CLP. The level of LDH was significantly higher in the CLP group than in the sham group at all timepoints (Figure 1D, *p* < 0.05), confirming successful establishment of the sepsis model. Cardiac function parameters, including CO, LVFS% and LVEF%, were markedly reduced in the CLP-12 h and CLP-24 h groups relative to the sham group (*p* < 0.001). CO, LVFS% and LVEF% continued to decline gradually in the CLP-36 h, CLP-48 h and CLP-72 h groups (Figure 1F–H, *p* < 0.01). Additionally, CK-MB level was increased obviously in the CLP-24 h group (Figure 1C, *p* = 0.0023). Western blot results demonstrated that LCN2 protein expression was markedly upregulated at 12 h and 24 h after CLP modeling (Figure 1I, *p* < 0.01).

In the HL-1 cardiomyocyte injury model, stimulation with LPS or RSL3 robustly increased cellular and mitochondrial ROS production (Figure 1K,M, *p* < 0.001). Intracellular Fe^2+^ accumulation was observed, accompanied by a prominent reduction in MMP (Figure 1R). Meanwhile, LCN2 protein expression was significantly increased (Figure 1P,T, *p* < 0.001), whereas the ferroptosis-related proteins GPX4 and DHODH were downregulated (Figure 1Q,U,V, *p* < 0.01).

### 2.2. Different Pathways of Ferroptosis Happened in Sepsis-Induced Cardiac Injury

**Figure 2:**
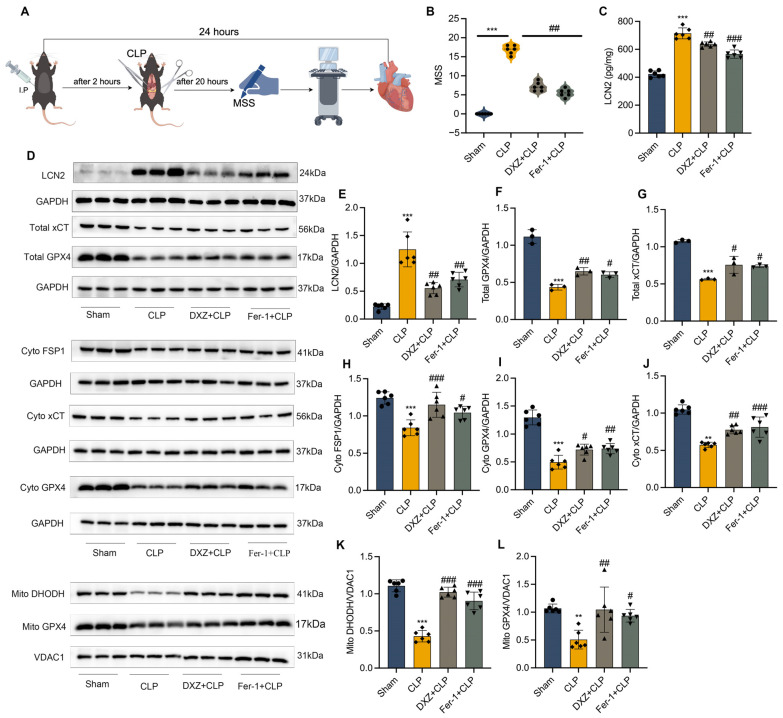
Different pathways of ferroptosis in sepsis-induced cardiac injury. (**A**): Experimental design drawing. (**B**): Murine sepsis score (MSS). (**C**): The myocardial LCN2 level in each group. (**D**): Representative images of the Western blot results. Changes in the protein expressions of LCN2 (**E**), Total GPX4 (**F**), Total xCT (**G**) (*n* = 3), Cyto FSP1 (**H**), Cyto xCT (**I**), Cyto GPX4 (**J**), Mito DHODH (**K**) and Mito GPX4 (**L**) (*n* = 6). *** *p* < 0.001, ** *p* < 0.01 vs. Sham group, ^###^ *p* < 0.001, ^##^ *p* < 0.01, ^#^ *p* < 0.05 vs. CLP group.

According to the above experimental findings, follow-up experiments were performed at 24 h post-CLP to characterize distinct ferroptosis pathways under septic stress. In the in vitro cardiomyocyte model, cells were treated with the iron chelator DXZ and canonical ferroptosis inhibitor Fer-1 to monitor ferroptotic alterations. The schematic experimental workflow is presented in Figure 2A. Western blot quantification of core myocardial ferroptosis-related proteins, including total GPX4, total xCT, cytosolic GPX4 (Cyto GPX4), cytosolic ferroptosis suppressor protein 1 (Cyto FSP1), cytosolic xCT (Cyto xCT), mitochondria-derived GPX4 (Mito GPX4) and mitochondria-derived DHODH (Mito DHODH), revealed simultaneous activation of multiple ferroptotic cascades in the CLP group (Figure 2D–L, *p* < 0.01–0.001). Treatment with iron chelator DXZ and ferroptosis inhibitor Fer-1 both alleviated sepsis severity, reflected by reduced murine sepsis score (MSS) values (Figure 2B, *p* < 0.01) and downregulated myocardial LCN2 protein abundance in CLP-operated mice (Figure 2C, *p* < 0.01).

### 2.3. Inhibiting Ferroptosis Decreased LCN2 Expression and Alleviate Myocardial Damage in Sepsis

**Figure 3:**
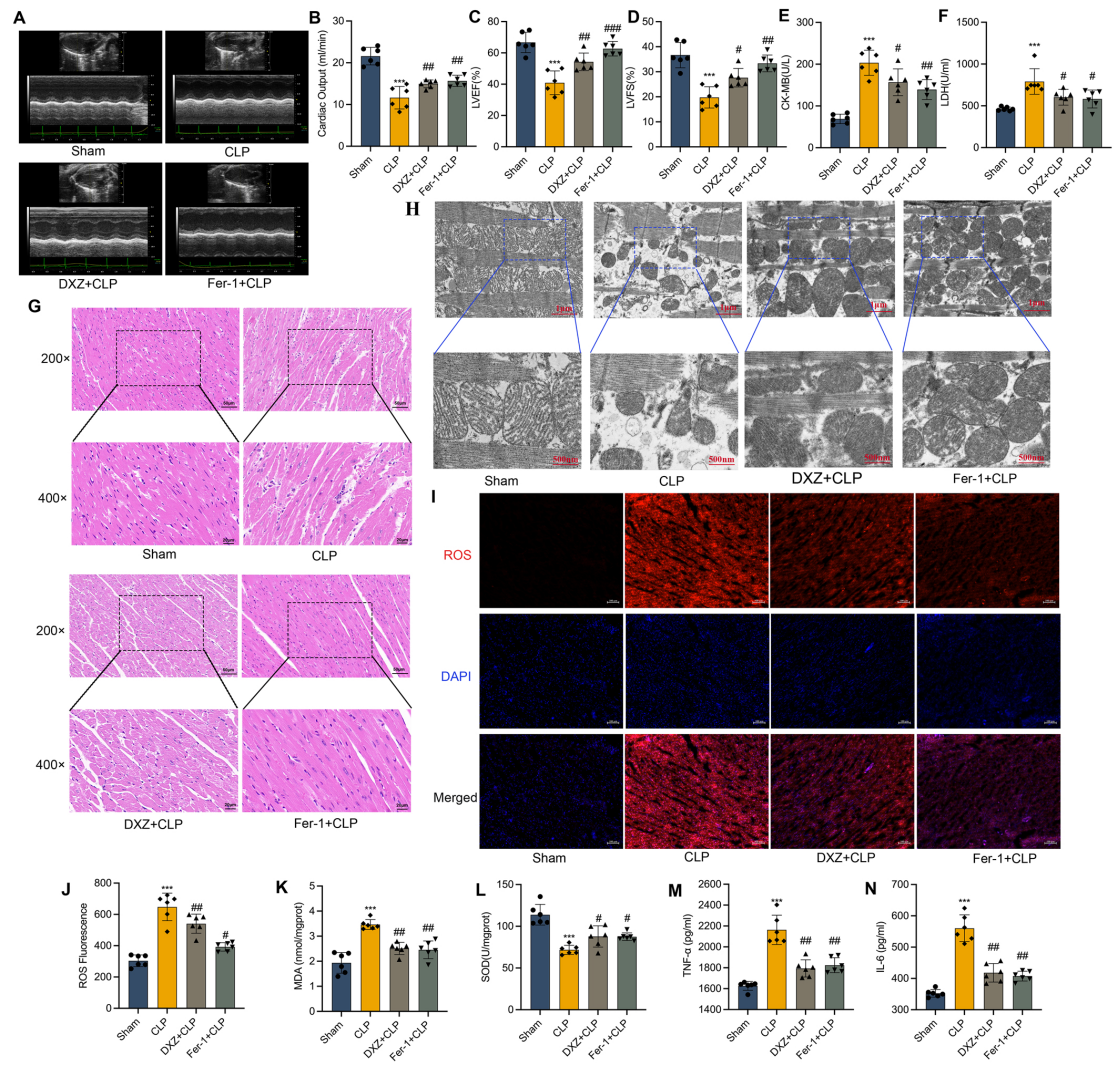
Inhibiting ferroptosis decreases LCN2 expression and alleviates myocardial damage in sepsis. (**A**,**G**,**H**,**I**): Representative images of echocardiographic M-mode (**A**), H&E (**G**), transmission electron microscopy (**H**) and fluorescence probe for ROS in cardiac tissues (**I**) (scale bar = 100 μm) in different groups. (**J**): The ROS fluorescence in each group. (**B**–**D**): Echocardiographic parameters. Cardiac output (**B**); left ventricle ejection fraction (**C**); left ventricular fractional shortening (**D**). (**E**,**F**): The serum CK-MB (**E**) and LDH (**F**) levels in each group. (**K**,**L**): Changes of myocardial MDA (**K**) and SOD (**L**) levels in different groups. (**M**,**N**): The serum TNF-α (**M**) and IL-6 (**N**) levels in each group. Data are presented as mean ± SD (*n* = 6). *** *p* < 0.001 vs. Sham group, ^###^ *p* < 0.001, ^##^ *p* < 0.01, ^#^ *p* < 0.05 vs. CLP group.

In the CLP group, HE staining also revealed that myocardial fibers were broken and cell infiltration was increased (Figure 3G). The levels of CK-MB and LDH were significantly decreased, with the increase in SOD level, suggesting that myocardial injury occurred in the CLP group, while DXZ (CK-MB: *p* =0.0243, LDH: *p* = 0.0273, SOD: *p* = 0.0339) and Fer-1 (CK-MB: *p* = 0.018, LDH: *p* = 0.0129, SOD: *p*= 0.0463) effectively reversed this myocardial injury (Figure 3E,F,L, *p* < 0.001–0.01). The serum levels of IL-6 and TNF-α in the CLP group were significantly higher than those in the sham group, whereas DXZ and Fer-1 treatment decreased the levels of IL-6 and TNF-α (Figure 3M,N, *p* < 0.001–0.01). The ferroptosis-related biochemical indicators MDA and ROS [38] were markedly elevated in CLP mice relative to sham mice (Figure 3K,J, *p* < 0.001). Typical mitochondrial morphological alterations were observed in the CLP group [39], including mitochondrial shrinkage, increased membrane density, and mitochondrial cristae disruption (Figure 3H). Both ELISA and Western blot analysis confirmed elevated LCN2 expression in cardiac tissue of CLP mice (Figure 2C,E, *p* < 0.001), and such upregulation was significantly reversed in the DXZ + CLP and Fer-1 + CLP groups. These results indicate that LCN2 expression is closely associated with ferroptosis in SIMI. Inhibiting ferroptosis reduced LCN2 expression and SIMI.

### 2.4. Knockdown of LCN2 Improves Mitochondrial Function and Alleviates Sepsis-Induced Ferroptosis

**Figure 4:**
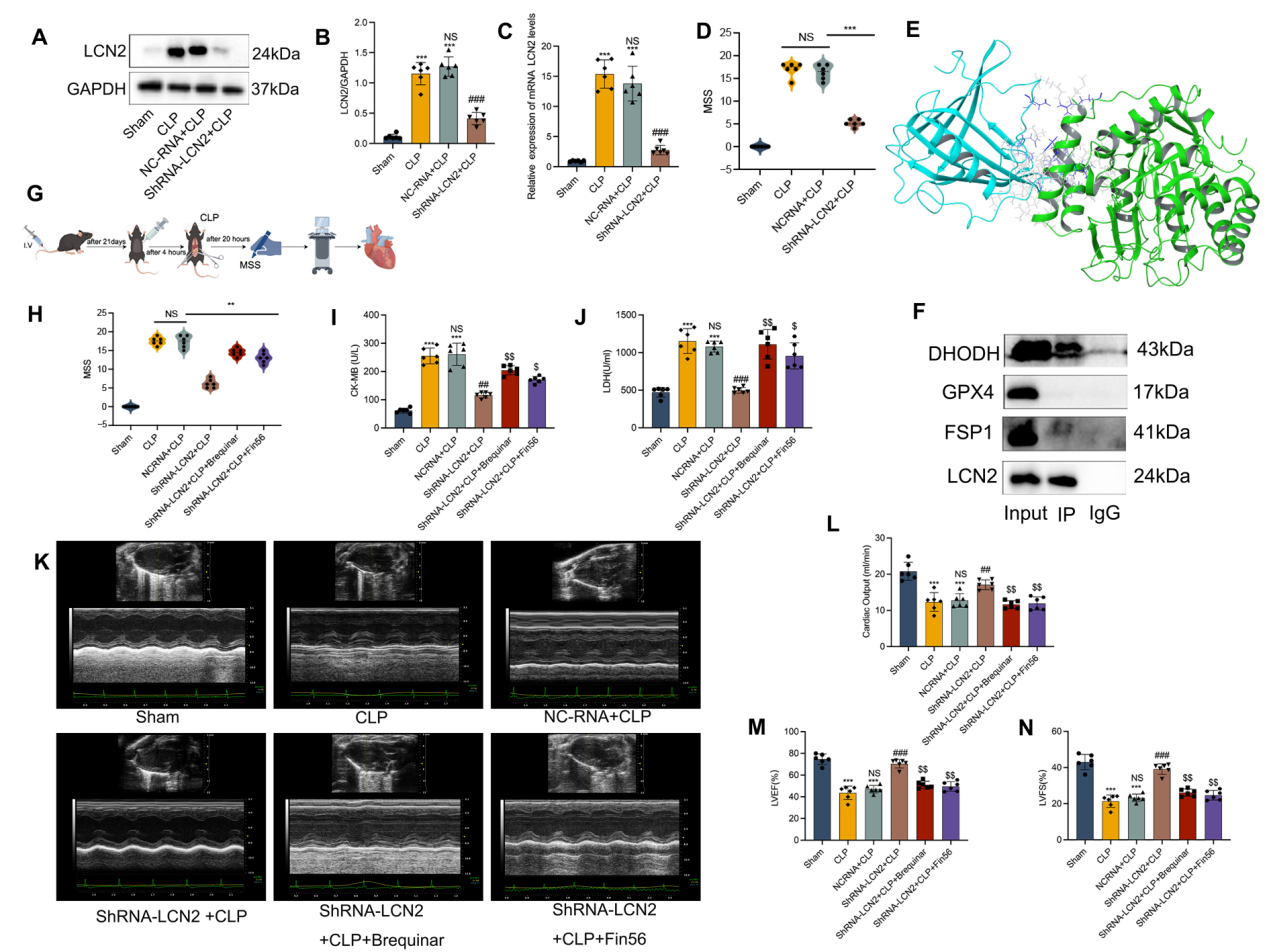
Knockdown of LCN2 improves mitochondrial function and alleviates sepsis-induced ferroptosis. (**A**): Representative images of Western blot results, (**E**): Molecular docking results (LCN2: green, DHODH: blue). (**F**): Immune-function precipitation results. Echocardiographic M-mode (**K**), H&E (**O**) and transmission electron microscopy (**P**) in cardiac tissues in different groups. (**B**): Changes in the protein expressions of LCN2 (*n* = 3). (**C**): The LCN2 level in mRNA expression was detected in each group by qPCR (*n* = 6). (**G**): Experimental design drawing. (**D,H**): Murine sepsis score. (**I**,**J**): The serum CK-MB (**I**) and LDH (**J**) levels in each group. Echocardiographic parameters: cardiac output (**L**); left ventricle ejection fraction (**M**); left ventricular fractional shortening (**N**). Data are presented as mean ± SD (*n* = 6). *** *p* < 0.001 vs. Sham group, ** *p* < 0.01 vs. NC-RNA + CLP group (**D**,**H**), ^###^ *p* < 0.001, ^##^ *p* < 0.01 vs. NC-RNA + CLP group, NS: *p* > 0.05 vs. CLP group, ^$$^ *p* < 0.01, ^$^ *p* < 0.05 vs. ShRNA-LCN2 + CLP group, ns: *p* > 0.05 vs. ShRNA-LCN2 + CLP group.

**Figure 5:**
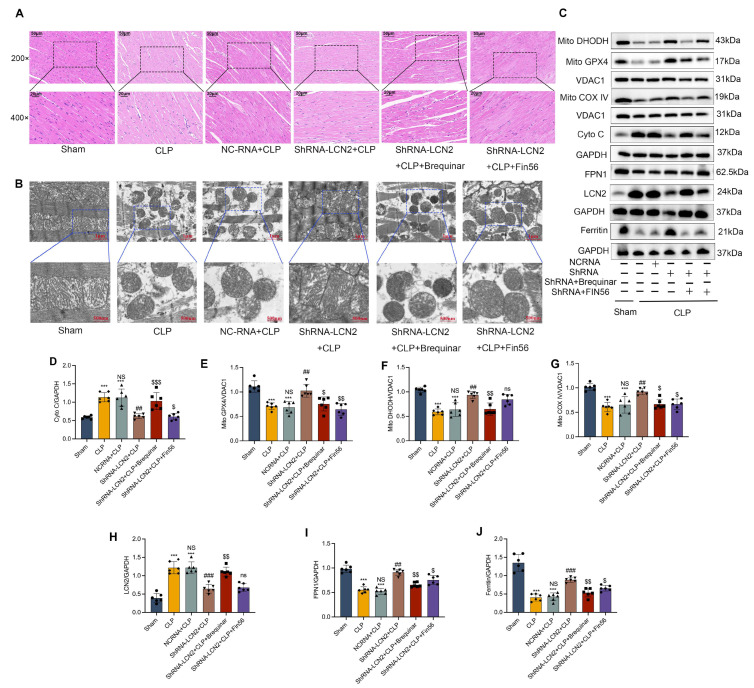
DHODH as a key protein of LCN2 affecting myocardial damage in sepsis. (**A**,**B**): Representative images of H&E (**A**) and transmission electron microscopy (**B**) in cardiac tissues in different groups. (**C**): Representative images of the Western blot results. Changes in the protein expressions of Cyto C (**D**), Mito GPX4 (**E**), Mito DHODH (**F**), Mito COX IV (**G**), LCN2 (**H**), FPN1 (**I**) and Ferritin (**J**). Data are presented as mean ± SD (*n* = 6). *** *p* < 0.001 vs. Sham group, ^###^ *p* < 0.001, ^##^ *p* < 0.01 vs. NC-RNA + CLP group, NS: *p* > 0.05 vs. CLP group, ^$$$^ *p* < 0.001, ^$$^ *p* < 0.01, ^$^ *p* < 0.05 vs. ShRNA-LCN2 + CLP group, ns: *p* > 0.05 vs. ShRNA-LCN2 + CLP group.

To assess the protective role of inhibiting LCN2 against SIMI, wild-type C57BL/6 mice received tail vein injections of AAV9-ShLCN2 at a dose of 2 × 10^11^ PFUs to achieve efficient LCN2 silencing. It was confirmed that myocardial LCN2 mRNA and protein abundances were markedly suppressed following AAV9-ShLCN2 administration (Figure 4A–C, *p* < 0.001). LCN2 knockdown significantly restored cardiac functional indicators (Figure 4 K–N, *p* < 0.001–0.01) and alleviated myocardial fiber lysis as well as inflammatory cell infiltration (Figure 5A). Ultrastructural observation revealed ameliorated mitochondrial morphological lesions (Figure 5B). Simultaneously, the mitochondrial biomarker COX IV was upregulated, while cytoplasmic cytochrome c (Cyto C) was downregulated (Figure 5D,G, *p* < 0.01). In addition, myocardial ROS accumulation was attenuated, accompanied by elevated expression of ferroptosis regulatory proteins, including ferritin, FPN1, mitochondrial GPX4 and mitochondrial DHODH (Figure 5I,J,E,F, *p* < 0.001–0.01). Collectively, LCN2 silencing confers myocardial protection against both cytosolic and mitochondrial ferroptosis under septic conditions.

### 2.5. DHODH as a Key Protein of Ferroptosis Against LCN2-Induced Myocardial Damage in Sepsis

**Figure 6:**
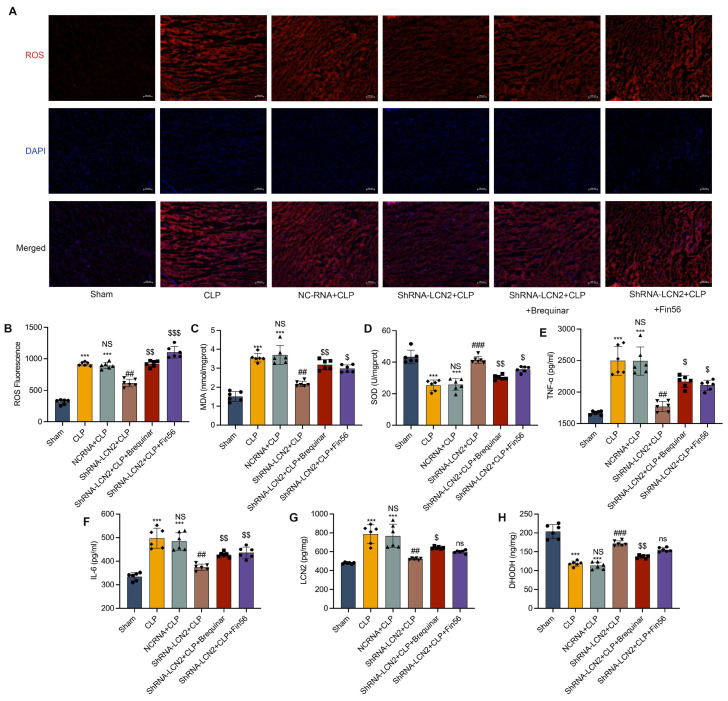
(**A**): Representative images of fluorescence probe for ROS in cardiac tissues (scale bar = 100 μm). (**B**): The ROS fluorescence in each group. (**C**,**D**): Changes of myocardial MDA (**C**) and SOD (**D**) levels in different groups. (**E**,**F**): The serum TNF-α (**E**) and IL-6 (**F**) levels in each group. (**G**,**H**): The cardiac LCN2 and DHODH levels in each group. Data are presented as mean ± SD (*n* = 6). *** *p* < 0.001 vs. Sham group, ^###^ *p* < 0.001, ^##^ *p* < 0.01 vs. NC-RNA + CLP group, NS: *p* > 0.05 vs. CLP group, ^$$$^ *p* < 0.001, ^$$^ *p* < 0.01, ^$^ *p* < 0.05 vs. ShRNA-LCN2 + CLP group, ns: *p* > 0.05 vs. ShRNA-LCN2 + CLP group. Notably, both inhibitors induced comparable myocardial ferroptotic injury, but only Brequinar upregulated p-STAT3, which indicates the specific interaction between the LCN2-DHODH axis and STAT3 signaling.

LCN2, an innate immune protein, has emerged as a critical iron regulatory protein under physiological and inflammatory conditions [40]. Through molecular docking and co-immunoprecipitation assays (docking source: LCN2-GPX4: −541.124, LCN2-FSP1: −544.532, LCN2-DHODH: −757.556), we verified the strong physical interaction between LCN2 and DHODH (Figure 4E,F). DHODH, a mitochondrial rate-limiting enzyme for de novo pyrimidine nucleotide synthesis, is indispensable for maintaining mitochondrial structural stability and the normal expression of mitochondrial functional proteins [10]. To dissect the regulatory linkage between LCN2 and mitochondrial ferroptosis, LCN2-knockdown mice received intraperitoneal injection of the specific DHODH inhibitor Brequinar (20 mg/kg) or mitochondrial GPX4 inhibitor Fin56 (10 mg/kg) prior to CLP surgery (Figure 4G). The results demonstrated that both Brequinar and Fin56 abolished the cardioprotective effect induced by LCN2 knockdown and aggravated myocardial ferroptotic injury. Brequinar treatment aggravated myocardial pathological damage and markedly upregulated LCN2 expression (Figure 5H, *p* < 0.001), whereas Fin56 did not exacerbate myocardial injury (Figure 5H, *p* = 0.9979) and increased sepsis severity scores (Figure 4H). Importantly, only Brequinar reversed the suppression of STAT3 phosphorylation caused by LCN2 silencing, whereas Fin56 did not affect p-STAT3 levels. Serum CK-MB, LDH, IL-6 and TNF-α concentrations, as well as myocardial MDA and ROS contents, were markedly elevated, while SOD was reduced (Figure 4I,G and Figure 6B–D, *p* < 0.001–0.01). ELISA and Western blot quantification revealed that, relative to the AAV9-ShLCN2 + CLP group, Brequinar treatment significantly downregulated cardiac DHODH and upregulated LCN2 expression; while Fin56 intervention caused no statistically meaningful alterations in LCN2 or DHODH protein levels. Ultrastructural observation revealed obvious mitochondrial shrinkage, thickened membrane density, disrupted cristae and partial outer membrane rupture in both AAV9-ShLCN2 + CLP + Brequinar and AAV9-ShLCN2 + CLP + Fin56 groups (Figure 5B). Consistently, mitochondrial COX IV expression was downregulated and cytoplasmic Cyto *c* was accumulated (Figure 5D,G). Collectively, these data illustrate that inhibition of DHODH or mitochondrial GPX4 abolishes the mitochondrial protective phenotypes mediated by LCN2 knockdown and aggravates SIMI, whereas mitochondrial GPX4 inhibition does not alter the expression of LCN2 or DHODH.

### 2.6. Recombinant LCN2 Protein Induces Cardiomyocyte Injury and Triggers Ferroptosis

**Figure 7:**
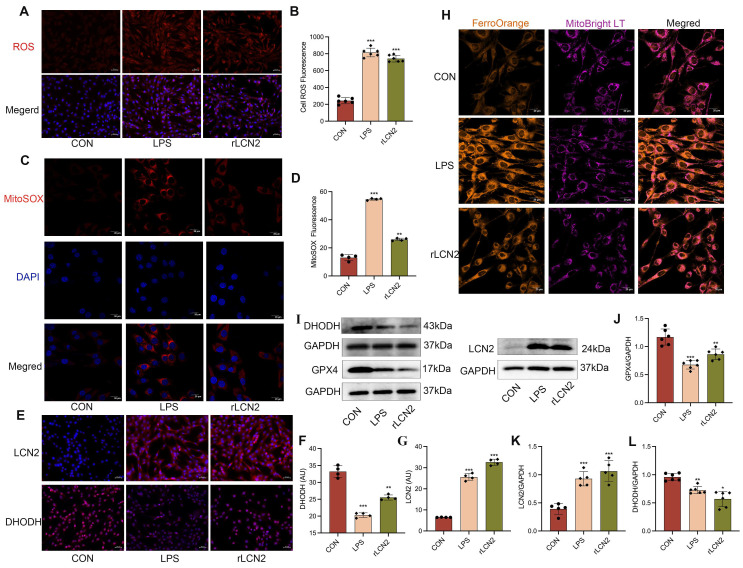
LCN2 induces ferroptosis in cardiomyocytes. (**A**): Representative images of fluorescence probe for ROS in HL-1 cardiomyocytes (*n* = 6, scale bar = 50 μm). (**B**): The ROS fluorescence in each group. (**C**): Images of mitochondrial ROS in different groups. (**D**): Mitochondrial ROS fluorescence intensity analysis. (*n* = 5, scale bar = 20 μm). (**E**): Images of LCN2 and DHODH (red) and DAPI (blue) immunofluorescence staining. (**F**,**G**): LCN2 and DHODH fluorescence intensity analysis. (*n* = 6, scale bar = 50 μm). (**H**): Detection of intracellular Fe^2+^ and mitochondrial morphology of HL-1 cardiomyocytes in different groups (scale bar = 20 μm). (**I**): Representative images of the Western blot results. Changes in the protein expressions of LCN2 (**K**), GPX4 (**J**) and DHODH (**L**) (*n* = 6). Data are presented as mean ± SD. *** *p* < 0.001, ** *p* < 0.01, * *p* < 0.05 vs. CON group.

To further investigate the potential regulatory effect of LCN2 on cardiomyocyte ferroptosis, we treated HL-1 cardiomyocytes with rLCN2. The results demonstrated that exogenous rLCN2 stimulation significantly promoted intracellular and mitochondrial ROS accumulation (Figure 7B,D), increased labile Fe^2+^ concentrations, and reduced MMP (Figure 7H). Meanwhile, rLCN2 intervention inhibited the expression of core ferroptosis regulators GPX4 and DHODH (Figure 7F,J,L), accompanied by a marked elevation in endogenous LCN2 protein abundance (Figure 7G,K).

### 2.7. Overexpression of DHODH Resists LPS-Induced Mouse Cardiomyocyte Injury

**Figure 8:**
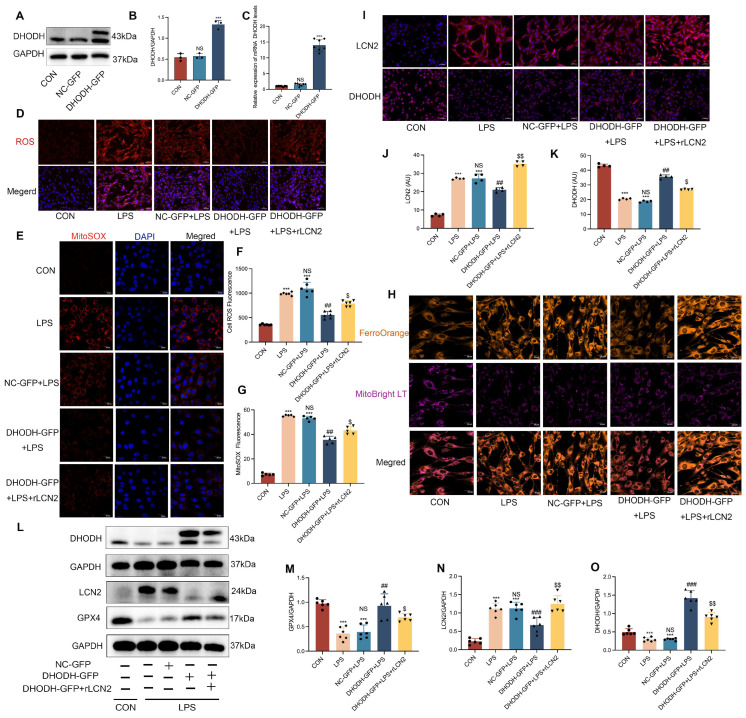
Overexpression of DHODH resists LPS-induced mouse cardiomyocyte injury. (**A**): Representative images of Western blots. (**B**): Changes in the protein expressions of DHODH (*n* = 3). (**C**): The LCN2 expression was detected by qPCR (*n* = 6). (**D**): Representative images of fluorescence probe for ROS in HL-1 cardiomyocytes (*n* = 6, scale bar = 50 μm). (**F**): The ROS fluorescence in each group. (**E**): Images of mitochondrial ROS in different groups. (**G**): Mitochondrial ROS fluorescence intensity analysis. (*n* = 5, scale bar = 20 μm). (**I**): Images of LCN2 and DHODH (red) and DAPI (blue) immunofluorescence staining. (**J**,**K**): LCN2 and DHODH fluorescence intensity analysis. (*n* = 6, scale bar = 50 μm). (**H**): Detection of intracellular Fe^2+^ and mitochondrial morphology of HL-1 cardiomyocytes in different groups (scale bar = 20 μm). (**L**): Representative images of the Western blot results. Changes in the protein expressions of GPX4 (**M**), LCN2 (**N**) and DHODH (**O**) (*n* = 6). Data are presented as mean ± SD. *** *p* < 0.001 vs. CON group. NS: *p* > 0.05 vs. CON group (Figure 8B,C), ^###^ *p* < 0.001, ^##^ *p* < 0.01 vs. NC-GFP + LPS group, NS: *p* > 0.05 vs. LPS group (Figure 8J,K,F,G,M,N,O), ^$$^ *p*< 0.01, ^$^ *p*< 0.05 vs. DHODH-GFP + LPS group.

To clarify whether DHODH serves as the core mediator of LCN2-driven ferroptosis under septic conditions, we constructed stable DHODH-overexpressing HL-1 cardiomyocytes via lentiviral transduction. Transfection efficiency was validated by qPCR and Western blot (Figure 8A–C, *p* < 0.001). In the LPS-stimulated cardiomyocyte injury model, DHODH overexpression markedly reduced total cellular and mitochondrial ROS accumulation, lowered labile intracellular Fe^2+^ concentration, and restored MMP (Figure 8H). Consistently, the protein abundances of GPX4, DHODH and FPN1 were significantly upregulated, whereas LCN2 expression was robustly suppressed (Figure 8M–O, *p* < 0.001–0.01). Moreover, rLCN2 administration fully abolished the cardioprotective phenotype conferred by DHODH overexpression in LPS-challenged HL-1 cells.

### 2.8. LCN2 Activates Ferroptosis Through Inhibiting DHODH Expression via STAT3 Signaling

**Figure 9:**
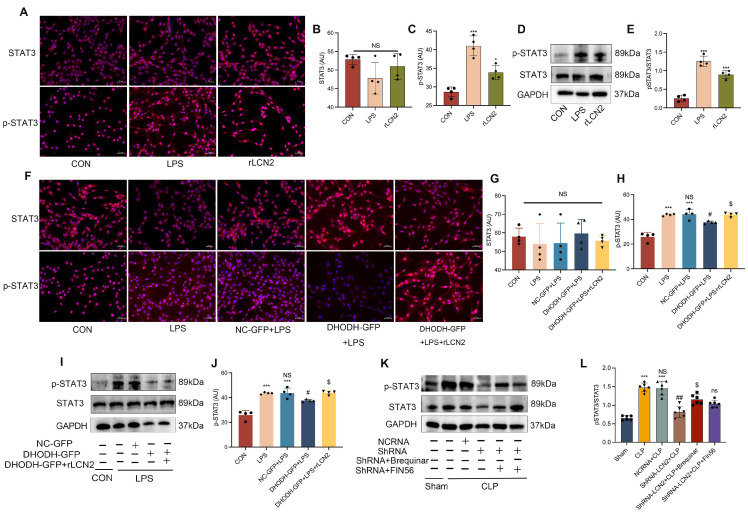
LCN2 activates ferroptosis by inhibiting DHODH expression via STAT3 signaling. (**A**,**F**): Images of STAT3 and p-STAT3 (red) and DAPI (blue) immunofluorescence staining. (**B**,**C**,**G**,**H**): STAT3 (**B**,**G**) and p-STAT3 (**C**,**H**) fluorescence intensity analysis. (*n* = 6, scale bar = 50 μm). (**D**,**I**,**K**): Representative images of the Western blot results. Changes in the protein expressions of P-STAT3 (**E**,**J**,**L**). Data are presented as mean ± SD (*n* = 6). *** *p* < 0.001, * *p* < 0.05 vs. CON group. NS: *p* > 0.05 vs. CON group (Figure 8B,C), ^##^ *p* < 0.01, ^#^ *p* < 0.05 vs. NC-GFP + LPS group, NS: *p* > 0.05 vs. LPS group (Figure 9H,J), ^$^ *p* < 0.05 vs. DHODH-GFP + LPS group (**B**,**C**,**E**,**G**,**H**,**J**). *** *p* < 0.001 vs. Sham group, ^##^ *p* < 0.01 vs. NC-RNA + CLP group, NS: *p* > 0.05 vs. CLP group, ^$^ *p* < 0.05 vs. ShRNA-LCN2 + CLP group, ns: *p* > 0.05 vs. ShRNA-LCN2 + CLP group (**L**).

The STAT3 signaling pathway participates in diverse biological processes. Existing evidence has confirmed that LCN2 triggers ferroptosis via STAT3 pathway activation [41], and STAT3 also exhibits tight functional crosstalk with DHODH to maintain mitochondrial homeostasis [42]. Therefore, we further investigated whether STAT3 mediates LCN2-driven septic myocardial injury. Immunofluorescence and Western blot data demonstrated that exogenous rLCN2 stimulation markedly elevated phosphorylated STAT3 (p-STAT3) levels in HL-1 cardiomyocytes (Figure 9A–E). In LPS-challenged DHODH-overexpressing HL-1 cells, rLCN2 treatment downregulated DHODH while concurrently upregulating p-STAT3 levels (Figure 9F–J). In vivo rescue experiments revealed that LCN2 silencing suppressed cardiac p-STAT3 accumulation; this inhibitory effect was fully abolished by the DHODH selective inhibitor Brequinar yet remained unchanged upon mitochondrial GPX4 inhibitor Fin56 intervention (Figure 9K,L).

## 3. Discussion

Sepsis is a life-threatening systemic inflammatory disorder, and myocardial dysfunction is a major determinant of high mortality in sepsis patients [43]. As terminally differentiated cells, cardiomyocytes suffer irreversible functional impairment following septic injury. Accumulating evidence confirms that cardiomyocyte death is a pivotal pathological driver of sepsis-induced myocardial injury (SIMI), making the suppression of aberrant cardiomyocyte death a promising therapeutic strategy for SIMI. Cardiomyocyte death encompasses apoptotic and non-apoptotic modalities, including necrosis, pyroptosis, and ferroptosis [44]. Abundant studies have demonstrated that ferroptosis is tightly implicated in diverse pathological processes and serves as a core mechanism underlying the progression of SIMI. Multiple independent but interactive metabolic pathways govern cellular ferroptosis [45]. Specifically, both cytosolic and mitochondrial GPX4 are central defenders against lipid peroxidation [46], FSP1 primarily mediates anti-ferroptotic effects at the plasma membrane [47] and DHODH exerts its regulatory functions in mitochondria [48]. Crosstalk among these distinct ferroptosis pathways collectively promotes myocardial oxidative damage and cardiac dysfunction. In the present study, pharmacological inhibition of ferroptosis using Fer-1 or the iron chelator DXZ markedly reduced myocardial LCN2 expression, alongside ameliorated oxidative stress and myocardial injury triggered by multi-pathway ferroptosis. Further elucidation of the molecular crosstalk between LCN2 and the ferroptotic cascade will provide novel insights for the optimization of SIMI prevention and treatment strategies.

LCN2 is a vital acute-phase protein that modulates multiple cellular biological processes and plays a pathogenic role in acute and chronic inflammation, tumors, neurological disorders, and metabolic diseases [49]. Clinically, LCN2 is a well-recognized sensitive biomarker for renal injury [50], and elevated plasma LCN2 independently predicts cardiac dysfunction and increased mortality in patients with severe sepsis and septic shock [51]. Mechanistically, LCN2 functions as a key iron transporter to maintain intracellular and extracellular iron homeostasis [28,52,53]. Given that iron metabolic balance is essential for normal cardiac function, LCN2 is indispensable for the regulation of cardiovascular iron homeostasis.

In vitro functional experiments in HL-1 cardiomyocytes demonstrated that LCN2 upregulated intracellular Fe^2+^ accumulation, enhanced cellular and mitochondrial ROS generation, and suppressed GPX4 activity, ultimately inducing oxidative stress and cardiomyocyte ferroptosis. Consistent with in vitro observations, LCN2 knockdown effectively mitigated myocardial ferroptosis and rescued SIMI in vivo. These findings robustly confirm that LCN2 exacerbates SIMI progression by promoting cardiomyocyte ferroptosis. Notably, our results revealed distinct phenotypic and molecular regulatory effects of LCN2: exogenous LCN2 stimulation induced mitochondrial dysfunction and activated STAT3 phosphorylation (p-STAT3) in HL-1 cells, while genetic ablation of LCN2 suppressed myocardial p-STAT3 activation in septic mice. Collectively, these data validate that LCN2 facilitates cardiomyocyte ferroptosis and SIMI progression via STAT3 signaling activation.

To further clarify the molecular mechanism by which LCN2 modulates ferroptosis in SIMI, molecular docking and co-immunoprecipitation (Co-IP) assays were performed to identify protein interaction profiles. The results verified that LCN2 specifically targets the mitochondrial DHODH-mediated ferroptosis pathway, rather than the classical GPX4 or FSP1-dependent ferroptosis pathways in SIMI. DHODH was successfully detected in LCN2-precipitated protein complexes, whereas GPX4 and FSP1 were undetectable, indicating a direct interaction between LCN2 and mitochondrial DHODH. DHODH is a mitochondrial rate-limiting enzyme responsible for de novo pyrimidine synthesis and has been identified as a therapeutic target for multiple malignancies [11,12,13,14,15,16]. Accumulating studies have demonstrated that DHODH inhibition suppresses tumor growth by triggering ferroptosis [10]. However, the regulatory relationship between DHODH and LCN2 in SIMI has not been previously reported. In vivo, LCN2 knockdown significantly upregulated myocardial DHODH expression, improved cardiac function, and alleviated lipid peroxidation, inflammatory infiltration, and oxidative stress in septic mice. In vitro, LPS stimulation induced LCN2 upregulation and concomitant DHODH downregulation in HL-1 cardiomyocytes. Moreover, exogenous recombinant LCN2 (rLCN2) treatment directly reduced DHODH levels and increased Fe^2+^ and ROS accumulation in cardiomyocytes. These findings confirm that LCN2 directly inhibits DHODH expression to initiate mitochondrial ferroptosis, whereas LCN2 deficiency elevates DHODH activity to exert prominent cardioprotective effects in sepsis.

Rescue experiments further revealed distinct regulatory effects of DHODH and GPX4 on STAT3 phosphorylation but consistent pro-ferroptotic phenotypic functions in SIMI. At the phenotypic level, both the DHODH inhibitor Brequinar and the GPX4 inhibitor Fin56 completely abrogated the cardioprotective effects induced by LCN2 knockdown and aggravated myocardial ferroptosis, showing identical pro-ferroptotic outcomes. However, at the molecular level, the two inhibitors exhibited totally different effects on STAT3 phosphorylation. Specifically, Brequinar treatment reversed the downregulated p-STAT3 level caused by LCN2 silencing and restored STAT3 activation, whereas Fin56 intervention produced no significant alteration in STAT3 phosphorylation. Given that DHODH is a critical component of the mitochondrial respiratory chain and participates in cellular energy metabolism and ATP production [54], we examined multiple mitochondrial-related indicators, including CYTO C and COX IV expression as well as ROS production. In LPS-stimulated HL-1 cells with DHODH overexpression, we observed decreased mitochondrial ROS levels and alleviated mitochondrial morphological damage, which suggests a protective effect on mitochondrial homeostasis. At the molecular level, p-STAT3 expression was significantly reduced. In contrast, exogenous rLCN2 treatment increased mitochondrial ROS accumulation, decreased MMP, and disrupted mitochondrial morphology, while upregulating p-STAT3 expression. The protective effects of DHODH overexpression were fully reversed by rLCN2 supplementation, accompanied by restored STAT3 phosphorylation. Consistent with in vitro results, Brequinar abolished the cardioprotective effects of LCN2 knockdown and restored STAT3 phosphorylation in septic mouse hearts, whereas Fin56 failed to alter p-STAT3 expression. Collectively, these results demonstrate a clear mechanistic distinction: LCN2 suppresses DHODH expression to enhance STAT3 phosphorylation at the molecular level, which subsequently triggers mitochondrial ferroptosis and ultimately exacerbates SIMI at the phenotypic level.

## 4. Materials and Methods

### 4.1. Experimental Animals

All specific pathogen-free (SPF) grade healthy C57BL/6 mice (male, 6–8 weeks old, weighing 18–22 g) were purchased from Jiangsu Qinglongshan Biological Technology Co., Ltd. (Zhenjiang, China), production license number SCXK (Su) 2024-0001. The animals were housed in an SPF-grade animal facility, maintained on a 12 h dark/light cycle, with ad libitum access to food and water. The temperature was maintained at 22–25 °C, and the humidity was kept at 60–70%. This study was approved by the Ethics Committee of Bengbu Medical College (Ethics number: [2022] No. 024), and all experiments complied with the “Regulations on the Administration of Laboratory Animals”.

### 4.2. Cecal Ligation and Puncture (CLP) Mouse Model

CLP is used in rodents to mimic the clinical course of sepsis [55]. The mice were anesthetized using a mixture of 3% isoflurane. After shaving and disinfecting the abdominal area, a midline incision was made with ophthalmic scissors. The cecum was ligated at the distal third with a 3-0 non-absorbable surgical suture and punctured twice at the distal end with an 18 G needle, gently squeezing out a small amount of feces. The cecum was then returned to the abdominal cavity and the incision was closed. In the sham operation group, the cecum was only exposed without ligation and puncture. Post-operatively, the mice were placed in a prone position and injected subcutaneously with pre-warmed 37 °C saline (10 mL/kg; s.c.) for fluid resuscitation. Before echocardiography, the severity of sepsis was assessed using the mouse sepsis score (MSS). The MSS method requires seven evaluation indicators: physical state, consciousness level, locomotor activity, response to external stimuli, ocular appearance, respiratory frequency, and breathing pattern. The composite score from these seven indicators represents the standardized MSS value [37]. After echocardiographic detection, mice were deeply anesthetized for retro-orbital venous blood sampling. Cardiac tissues were harvested immediately after blood collection and stored at −80 °C for subsequent biochemical and molecular analyses.

### 4.3. Survival Analysis

Survival analysis was performed in an independent mouse cohort, and no animals in this survival experiment were reused for biochemical, molecular, or histological detection, thereby avoiding the interference of early mortality on subsequent experimental results. Mice were randomly divided into the sham group and CLP group. The survival status of mice was monitored every 12 h for 7 consecutive days after surgery. Survival curves were plotted via the Kaplan–Meier method, and the log-rank (Mantel–Cox) test was applied to evaluate statistical differences in survival rates between groups. All survival analyses were performed using GraphPad Prism 9 (GraphPad Software, La Jolla, CA, USA).

### 4.4. Construction of shRNA and Lentiviral Vectors

Short hairpin RNA (shRNA) targeting mouse lipocalin-2 (LCN2) was synthesized by Genechem Co., Ltd. (Shanghai, China) to suppress endogenous LCN2 expression. The targeting sequence of LCN2 shRNA was as follows: 5′-GCTTTACGATGTACAGCACCA-3′. Mice were administered tail vein injections of either AAV9-shLCN2 or control AAV9-EGFP-NC for 3 weeks prior to CLP surgery to silence myocardial LCN2 expression.

Lentiviral vectors carrying DHODH-EGFP were constructed to establish stable DHODH-overexpressing HL-1 cardiomyocyte lines. Mouse HL-1 cardiomyocytes were infected with DHODH-EGFP lentivirus or negative control NC-EGFP lentivirus. Successfully infected positive cells were screened with 2.5 μg/mL puromycin for 2 weeks post-infection. Transduction efficiency was validated via Western blot and quantitative real-time PCR (qRT-PCR).

### 4.5. Pharmacological Intervention

All in vivo pharmacological interventions were performed on CLP-induced septic mice, with corresponding vehicle control groups injected with equal volumes of DMSO solvent. Briefly, mice were intraperitoneally injected with 50 mg/kg DXZ or 5 mg/kg Fer-1 at 2 h before CLP surgery to induce or inhibit myocardial ferroptosis [30]. For LCN2 loss-of-function rescue experiments, mice received tail vein injection of AAV9-ShRNA-LCN2 21 days prior to CLP modeling, followed by intraperitoneal administration of 20 mg/kg DHODH inhibitor Brequinar or 10 mg/kg mitochondrial GPX4 inhibitor Fin56 at 4 h before surgery to regulate mitochondrial ferroptosis [48]. All compounds were dissolved in consistent DMSO vehicle for uniform delivery. The dosages and pre-treatment timepoints of DXZ, Fer-1, Brequinar and Fin56 were validated by our internal pilot animal assays and consistent with well-established published protocols, which guarantee effective on-target binding and stable pharmacological effects on their respective ferroptosis-related targets. All agents were given as a single pre-operative injection, and their target engagement and specific inhibitory/inductive activity have been thoroughly characterized in previous mechanistic studies.

### 4.6. Quantification of Cardiac Injury and Functional Assessment In Vivo

The echocardiographic examination was performed in vivo at 24 h post-CLP using the Vevo-2100 imaging system (FujiFilm VisualSonics, Toronto, ON, Canada). Cardiac function was evaluated by detecting the left ventricular ejection fraction (LVEF%), fractional shortening (LVFS%) and cardiac output (CO).

To quantify myocardial injury in mice [56,57], serum creatine kinase-MB (CK-MB) and lactate dehydrogenase (LDH) concentrations were determined with corresponding commercial assay kits (E006-1-1, Nanjing Jiancheng Bioengineering Institute, Nanjing, China and BC0685, Solarbio, Beijing, China respectively).

### 4.7. Determination of MDA and SOD Levels

According to the manufacturer’s instructions, malondialdehyde (MDA) and superoxide dismutase (SOD) concentrations in cardiac tissue were determined using the corresponding commercial assay kit (A003-1 and A001-3, Nanjing Jiancheng Bioengineering Institute, Nanjing, China).

### 4.8. Enzyme-Linked Immunosorbent Assay (ELISA) Analysis

According to the manufacturer’s protocols, the concentrations of serum inflammatory cytokines IL-6 and TNF-α, as well as myocardial LCN2 and DHODH, were quantified via commercial ELISA kits. The corresponding assay kits were purchased from Jiangsu Jingmei BioTech Co., Ltd. (Yancheng, China): mouse IL-6 (JM-02446M1), TNF-α (JM-02415M1), LCN2 (JM-12543M1), and DHODH (JM-12822M).

### 4.9. Hematoxylin and Eosin (H&E) Staining Observation

For histological analysis, the harvested cardiac tissues were fixed in 4% paraformaldehyde for 72 h, dehydrated, cleared, paraffin-embedded, and cut into 6 μm thick sections for hematoxylin and eosin (H&E) staining. Morphological alterations including inflammatory infiltration and collagen deposition were observed under an optical microscope (Nikon ECLIPSE E100, Tokyo, Japan) to evaluate myocardial pathological injury.

### 4.10. Transmission Electron Microscopy (TEM) Observation

The myocardial tissue (≤1 mm^3^) was rapidly dissected from the left ventricle and rinsed with pre-cooled PBS. Subsequently, tissues were immediately fixed overnight at 4 °C in phosphate-buffered 2.5% glutaraldehyde solution (pH 7.4). Post-fixation processing and embedding were performed, after which ultrathin sections (50 nm) were cut, stained with uranyl acetate and lead citrate, and visualized under a transmission electron microscope (HITACHI HT7800, Tokyo, Japan).

### 4.11. Detection of ROS Levels in Myocardial Tissue Using DHE Fluorescent Probe

The myocardial tissue was washed with PBS (pH 7.4) and placed in embedding molds. Embedding medium was immediately added, and the samples were frozen for sectioning. The tissue was sectioned into 6 μm thick slices using a cryostat and mounted on adhesion slides. The sections were incubated in a working solution of 10 µmol/L dihydroethidium (DHE) in a water bath at 37 °C for 30 min in the dark. Following incubation, the nuclei were counterstained with 5 μg/mL DAPI working solution. The changes in fluorescence intensity were observed using an inverted fluorescence microscope (Zeiss, Obsever Z1, Oberkochen, Germany).

### 4.12. RNA Extraction Using TRIzol and Quantitative Real-Time Polymerase Chain Reaction (qPCR)

Total RNA was extracted from cardiac tissue and cultured HL-1 cardiomyocytes using TRIzol reagent (15596026, Invitrogen, Carlsbad, CA, USA). The purified RNA was then reverse-transcribed into complementary DNA (cDNA) with the FastKing First-Strand cDNA Synthesis Kit (KR118, Tiangen Biotech, Beijing, China). qRT-PCR was conducted using SuperReal PreMix Plus (FP205, Tiangen Biotech, Beijing, China). Target gene expression was normalized to the reference gene GAPDH to calculate relative expression levels, and fold changes were quantified via the 2^−ΔΔCT^ method. Sequences of all primers used in this study are listed in Table 1.

### 4.13. Molecular Docking Analysis and Co-Immunoprecipitation (Co-IP) to Observe the Relationship Between LCN2 and Ferroptosis-Related Proteins

For predicting the direct binding models of LCN2 protein with GPX4, FSP1, and DHODH, we first retrieved detailed information including structural files and sequence data from the Uniprot database for LCN2, GPX4, and DHODH. The corresponding PDB IDs were as follows: LCN2: 3T1D, GPX4: 6hkq, DHODH: 7Z6C. We utilized the AlphaFold 2 protein structure prediction tool to generate the full-length structural model of FSP1.

All protein structures were processed using UCSF Chimera (version 1.17.1) software: hydrogen atoms were added, partial charges were computed and assigned, and energy minimization was performed. Protein–protein molecular docking simulations were carried out using ClusPro 2.0 web server (https://cluspro.bu.edu, accessed 1 March 2023) software. After docking, LCN2-containing complex structures with the most favorable binding energies were visualized and further refined using Maestro (version 13.5, Schrödinger, LLC, New York, NY, USA).

For Co-IP assays, Protein A + G magnetic beads (P2108-5, Beyotime, Shanghai, China) were transferred into EP tubes assigned to the IP and IgG groups. The beads were rinsed with PBS via gentle inversion, separated on a magnetic rack to discard supernatants, and this washing cycle was repeated twice. Following the manufacturer’s guidelines, LCN2 primary antibody was added to IP tubes, whereas an equivalent volume of normal IgG antibody (A7007, Beyotime, Shanghai, China) was added to the negative control tubes. The mixtures were incubated on a horizontal shaker at 4 °C for 12 h. After incubation, the magnetic beads were washed three times, followed by supplementation of total protein lysates from CLP-operated mice into each tube; samples were incubated on a shaker at 4 °C for another 12 h. Input positive control, IP, and IgG negative control samples were denatured in 3× protein loading buffer at 95 °C for 5 min, cooled to 4 °C, and subjected to Western blot to detect physical interactions between LCN2 and GPX4 and DHODH as well as FSP1.

### 4.14. Mouse HL-1 Cell Culture

HL-1 cardiomyocytes were cultured in Claycomb medium (iCell-0012, Shanghai iCell, Shanghai, China) supplemented with 10% FBS at 37 °C in a humidified atmosphere containing 5% CO_2_ in a CO_2_ incubator (HF100, Thermo Fisher Scientific, Waltham, MA, USA). To verify LPS-triggered ferroptosis, HL-1 cells were exposed to 20 μg/mL LPS (L3024, Sigma-Aldrich, St. Louis, MO, USA) for 48 h after screening the optimal concentration via cell viability analysis [31]. Aside from the control group, cells in the ferroptosis positive group were incubated with 10 μM RSL3 (SML2234, Sigma-Aldrich, St. Louis, MO, USA) for 24 h. To investigate the correlation between LCN2 and ferroptosis, cells were stimulated with 1 μg/mL recombinant LCN2 protein (rLCN2) (CM17, Novoprotein, Shanghai, China) for 24 h [58]. To further clarify the regulatory link between LCN2 and DHODH under septic conditions, DHODH-overexpressing HL-1 cells were co-treated with 1 μg/mL rLCN2 and LPS for 24 h.

### 4.15. Analysis of Cell Viability and LDH Levels

Cell viability was assessed using the Cell Counting Kit-8 (CCK-8) (BS350A, Biosharp, Hefei, China) after different treatments. HL-1 cardiomyocytes were seeded into 96-well plates and incubated with CCK-8 working solution (100 μL per well diluted in complete medium) at 37 °C in a 5% humidified CO_2_ incubator for 1 h. Optical density values were detected at 450 nm with a microplate reader (BioTek, Winooski, VT, USA).

### 4.16. Cell ROS Measurement by DHE

The cells were washed twice with pre-warmed PBS. Following the manufacturer’s instructions, 10 μM DHE working solution (S0063, Beyotime, Shanghai, China) diluted in MEM medium was applied to the cells. The cells were then incubated in a humidified 5% CO_2_ incubator at 37 °C for 30 min in the dark. Subsequently, cells were fixed with 4% paraformaldehyde at room temperature for 20 min. After permeabilization with permeabilization buffer at room temperature for 10 min, cells were incubated with 5 μg/mL DAPI working solution (S0063, Beyotime, Shanghai, China) at 37 °C for 7 min in the dark to counterstain the nuclei. Images were captured using a Zeiss inverted fluorescence microscope (Zeiss, Observer Z1, Germany).

### 4.17. Mitochondrial ROS Measurement by MitoSOX

The cells were rinsed twice with pre-warmed Hanks’ balanced salt solution (HBSS) (BL561A, Biosharp, Hefei, China). Cells were incubated with 1 mL MEM medium containing 5 μM MitoSOX fluorescent probe (M36008, Thermo Fisher Scientific, Waltham, MA, USA) at 37 °C for 30 min. Then, cells were washed three times with PBS following the manufacturer’s protocols. Subsequently, cells were fixed in pre-warmed 4% paraformaldehyde at room temperature for 20 min. After permeabilization with permeabilization buffer (P0097, Beyotime, Shanghai, China) for 10 min at room temperature, cell nuclei were counterstained with 5 μg/mL DAPI working solution. Mitochondrial ROS signals were detected via a two-photon laser scanning confocal microscope (FV-1200MPE-SHARE, Olympus, Tokyo, Japan). FlowJo and GraphPad Prism 9 software were used for quantitative analysis.

### 4.18. Mitochondrial Imaging and Fe^2+^ Content Measurement

To verify mitochondrial injury induced by LPS, RSL3 and rLCN2, as well as the protective function of DHODH, we performed mitochondrial staining and assessed mitochondrial membrane potential (MMP). Cells were rinsed twice with pre-warmed HBSS. According to the manufacturer’s instructions, 1 mL MEM medium supplemented with 0.1 μmol/L MitoBright LT Deep Red fluorescent probe (MT12, Dojindo, Mashiki, Japan) was added, and cells were incubated at 37 °C for 15 min in a light-proof incubator. After incubation, cells were washed three times with pre-warmed HBSS. Then, cells were treated with HBSS containing 1 μmol/L Fe^2+^ probe (F374, Dojindo, Mashiki, Japan) and incubated at 37 °C in a 5% CO_2_ incubator for 30 min. The MMP level and intracellular Fe^2+^ concentration was detected using a two-photon laser scanning confocal microscope (FV-1200MPE-SHARE, Olympus, Hachioji City, Japan). Quantification analysis was conducted with FlowJo and GraphPad Prism 9 software.

### 4.19. Immunofluorescence (IF)

The cells were washed 3 times using PBS and fixed with 4% paraformaldehyde (BL539A, Biosharp, Hefei, China) at room temperature for 20 min. Immunostaining permeabilization buffer was applied for 10 min, followed by blocking with 5% bovine serum albumin (BSA, 143183, Germany Biofroxx, Einhausen, Germany) at 37 °C for 90 min. The cells were then incubated overnight at 4 °C with rabbit anti-LCN2 antibody (26991-1-AP, China Proteintech, Wuhan, China), anti-DHODH (14877-1-AP, China Proteintech, Wuhan, China), STAT3 antibody (AF6294, USA Affinity, Newtown Square, PA, USA), and p-STAT3 antibody (AF3293, USA Affinity, Newtown Square, PA, USA). After washing three times with PBS, cells were incubated with corresponding goat anti-rabbit IgG-Alexa Fluor secondary antibody (ZF-0516, Beijing Zhongsheng Golden Bridge Biotechnology Co., Ltd., Beijing, China) diluted 1:200 at 37 °C for 30 min. DAPI staining (C1002, Beyotime, Shanghai, China) was performed for 7 min. Images were acquired using a Zeiss fluorescence inverted microscope (Zeiss, Observer Z1, Oberkochen, Germany).

### 4.20. Western Blotting

Mitochondria were isolated from fresh cardiac tissue using the Tissue Mitochondria Isolation Kit (C3606, Beyotime, Shanghai, China) following the manufacturer’s instructions. Cytoplasmic fractions were simultaneously collected. Total proteins from cardiac tissues and HL-1 cardiomyocytes were extracted with RIPA lysis buffer (P0013B, Beyotime, Shanghai, China) containing protease and phosphatase inhibitor cocktails, followed by 30 min lysis on ice. The lysates were centrifuged at an appropriate speed at 4 °C for 15 min to collect supernatants. The BCA protein quantification kit was used to measure total protein concentrations of supernatants. Protein samples (20 μg per lane) were separated on 10% SDS-PAGE gels and subsequently transferred onto PVDF membranes (IPVH0001, Merck Millipore, Burlington, MA, USA). PVDF membranes were blocked with non-protein blocking buffer (PS108, Shanghai Yenzyme Biotech, Shanghai, China) at room temperature for 30 min. Membranes were incubated overnight at 4 °C with the following primary antibodies: GAPDH (1:7000, abs132004, Absin, Shanghai, China), LCN2 (1:1000, AF1857, R&D Systems, USA), GPX4 (1:3000, ab125066, Abcam, Waltham, MA, USA), xCT (1:5000, ab175186, Abcam, Waltham, MA, USA), Ferritin (1:1000, ab75973, Abcam, Waltham, MA, USA), FPN1 (1:2000, NBP1-21502, NOVUS, USA), COX IV (1:2000, WL02203, Wanlei Bio, Shenyang, China), Cytochrome c (1:2000, WL02410, Wanlei Bio, Shenyang, China), STAT3 (1:1000, AF6294, Affinity, San Francisco, CA, USA), Phospho-STAT3-Tyr705 (1:1000, AF3293, Affinity, San Francisco, CA, USA). After washing with Tris-buffered saline with Tween 20 (TBST) 3 times for 5 min each, membranes were incubated with appropriate horseradish-peroxidase-conjugated secondary antibodies (1:5000, Affinity, San Francisco, CA, USA) for 30 min at 4 °C. Protein bands were visualized using a highly sensitive enhanced chemiluminescence (ECL) substrate (P90720, Millipore, Burlington, MA, USA). The gray intensity of each band was quantified via Fiji (ImageJ version 1.54) software. The relative expression of total/cytosolic proteins was normalized to GAPDH, while mitochondrial protein expression was normalized to VDAC1.

### 4.21. Statistical Analysis

Survival analysis was performed using an independent mouse cohort; no animals in the survival cohort were reused for subsequent biochemical, histological, or molecular experiments. The Kaplan–Meier method and log-rank test were adopted to assess intergroup survival disparities. All quantitative results were expressed as mean ± SD. At least three independent biological replicates were carried out for cellular experiments, and each animal group contained *n* ≥ 3–6 samples. Prior to parametric statistical analysis, the Shapiro–Wilk test was used for normality verification, and Levene’s test was performed to assess the homogeneity of variance. Data conforming to a normal distribution and equal variance were analyzed by one-way ANOVA followed by Tukey’s post hoc test with rigorous multiple-comparison correction. Non-normally distributed data were analyzed using the Kruskal–Wallis H test with Dunn’s multiple comparison correction. For longitudinal multi-timepoint cardiac function data, repeated-measures one-way ANOVA with Bonferroni post hoc correction was applied. All statistical analyses and graphical visualization were performed using GraphPad Prism 9. Statistical significance was defined as *p* < 0.05, and NS indicates no significant difference (*p* > 0.05).

## 5. Conclusions

LCN2 drives mitochondrial ferroptosis during sepsis-induced myocardial injury. LCN2 deficiency increases DHODH expression and inhibits STAT3 phosphorylation, thereby mitigating SIMI. This study reveals the binding interaction between LCN2 and DHODH in septic myocardium and provides a new target for SIMI intervention.

## Figures and Tables

**Table 1:** Sequences of primer pairs used for quantitative real-time PCR analysis.

Target Gene	Primer Sequence (5′→3′)		Product Length (bp)	NCBI Accession No.
Lcn2	Forward: TGGCCCTGAGTGTCATGTG	Reverse: CTCTTGTAGCTCATAGATGGTGC	239	NM_008491.4
Dhodh	Forward: TCTTCACCTCTTACCTGACAGC	Reverse: CATGTTGGAGTCCTGAAACGTA	164	NM_010057.3
Gapdh	Forward: AGGTCGGTGTGAACGGATTTG	Reverse: TGTAGACCATGTAGTTGAGGTCA	123	NM_008084.4

## Data Availability

The original contributions presented in this study are included in the article. Further inquiries can be directed to the corresponding author.

## Abbreviations

The list of abbreviations:

Abbreviation	Full Name
CLP	cecal ligation and puncture
CO	cardiac output
Co-IP	co-immunoprecipitation
COX IV	cytochrome c oxidase subunit IV
Cyto C	cytochrome *c*
DHE	dihydroethidium
DHODH	dihydroorotate dehydrogenase
DXZ	dexrazoxane
ELISA	enzyme-linked immunosorbent assay
ETC	electron transport chain
Fe^2+^	ferrous ions
Fer-1	ferrostatin-1
FPN1	ferroportin 1
FSP1	ferroptosis suppressor protein 1
GPX4	glutathione peroxidase 4
HBSS	Hanks’ balanced salt solution
IF	immunofluorescence
LCN2	lipocalin-2
LPS	lipopolysaccharide
LVEF	left ventricular ejection fraction
LVFS	left ventricular fractional shortening
MDA	malondialdehyde
MMP	mitochondrial membrane potential
MODS	multiple-organ dysfunction syndrome
PLOOHs	phospholipid hydroperoxides
p-STAT3	phosphorylated STAT3
qPCR	quantitative PCR
rLCN2	recombinant LCN2 protein
ROS	reactive oxygen species
SIMI	sepsis-induced myocardial injury
SOD	superoxide dismutase
STAT3	signal transducer and activator of transcription 3
TEM	transmission electron microscopy
